# Supplementary material for: Cognitive ability and voting behaviour in the 2016 UK referendum on European Union membership
Source: PLoS One. 2023 Nov 22;18(11):e0289312. doi: 10.1371/journal.pone.0289312 (PMC10664886; doi:10.1371/journal.pone.0289312)
Supplement: S1 Table — (DOCX) [file pone.0289312.s001.docx]

**Table S1.** **Descriptive statistics**

|  | *Mean/*  *Frequency* | *Std. Dev.* | *Min* | *Max* |
| --- | --- | --- | --- | --- |
|  |  |  |  |  |
| Voted Remain | 0.566 | 0.496 |  |  |
| Word Recall | 12.35 | 3.073 | 0 | 20 |
| Verbal Fluency | 23.99 | 6.309 | 0 | 50 |
| Subtraction Test | 4.649 | 0.798 | 0 | 5 |
| Fluid Reasoning | 539.0 | 26.35 | 409 | 584 |
| Numerical Reasoning | 4.010 | 0.933 | 0 | 5 |
| Age (years) | 53.56 | 13.28 | 21 | 89 |
| Male | 0.500 |  |  |  |
| White | 0.921 |  |  |  |
| University or college degree | 0.366 |  |  |  |
| Other higher degree | 0.140 |  |  |  |
| A-Level | 0.180 |  |  |  |
| GCSE's | 0.177 |  |  |  |
| Other qualification | 0.0811 |  |  |  |
| No qualification | 0.0559 |  |  |  |
| Employee | 0.537 |  |  |  |
| Self-employed | 0.0990 |  |  |  |
| Unemployed | 0.0123 |  |  |  |
| Full-time education | 0.00220 |  |  |  |
| Retired | 0.293 |  |  |  |
| Economically inactive | 0.0562 |  |  |  |
| Interview mode: Face to face | 0.198 |  |  |  |
| Interview mode: Telephone | 0.0463 |  |  |  |
| Interview mode: Web | 0.756 |  |  |  |
| Number of sources for news | 3.857 | 2.009 | 0 | 12 |
| Doesn't read a newspaper | 0.398 |  |  |  |
| Broadsheets | 0.249 |  |  |  |
| Redtop tabloids | 0.0975 |  |  |  |
| Compact tabloids | 0.143 |  |  |  |
| Compact newspapers | 0.0162 |  |  |  |
| Regional newspapers | 0.0961 |  |  |  |
| Not a political supporter | 0.155 |  |  |  |
| Conservative | 0.337 |  |  |  |
| Labour | 0.269 |  |  |  |
| Liberal | 0.0748 |  |  |  |
| Green | 0.0388 |  |  |  |
| Nationalist | 0.123 |  |  |  |
| Other political party | 0.00314 |  |  |  |
| General health: Excellent | 0.179 |  |  |  |
| General health: Very good | 0.405 |  |  |  |
| General health: Good | 0.279 |  |  |  |
| General health: Fair | 0.112 |  |  |  |
| General health: Poor | 0.0243 |  |  |  |
| Long-term health condition | 0.329 |  |  |  |
| Openness | 4.648 | 1.169 | 1 | 7 |
| Neuroticism | 3.490 | 1.330 | 1 | 7 |
| Extraversion | 4.576 | 1.264 | 1 | 7 |
| Conscientious | 5.589 | 0.958 | 1 | 7 |
| Agreeableness | 5.592 | 0.932 | 1.333 | 7 |
| Log of OECD household income (deflated) | 7.725 | 0.556 | 1.751 | 9.661 |
| Married | 0.899 |  |  |  |
| Number of children in household | 0.594 | 0.940 | 0 | 6 |
| Square root of household size | 1.688 | 0.308 | 1.414 | 3.162 |
| Own house outright | 0.447 |  |  |  |
| Own house with mortgage | 0.461 |  |  |  |
| Private sector renter | 0.0515 |  |  |  |
| Local authority renter | 0.0412 |  |  |  |
| Financial decision maker: Respondent | 0.113 |  |  |  |
| Financial decision maker: Spouse | 0.126 |  |  |  |
| Financial decision maker: Equal | 0.760 |  |  |  |
| Financial decision maker: Other | 0.000785 |  |  |  |
| Lives in urban area | 0.698 |  |  |  |
| North East | 0.0443 |  |  |  |
| North West | 0.108 |  |  |  |
| Yorkshire and Humber | 0.0723 |  |  |  |
| East Midlands | 0.0814 |  |  |  |
| West Midlands | 0.0792 |  |  |  |
| East of England | 0.104 |  |  |  |
| London | 0.0644 |  |  |  |
| South East | 0.146 |  |  |  |
| South West | 0.104 |  |  |  |
| Wales | 0.0600 |  |  |  |
| Scotland | 0.0964 |  |  |  |
| Northern Ireland | 0.0386 |  |  |  |
|  |  |  |  |  |
| Number of individuals | 6,366 |  |  |  |
| Number of households | 3,183 |  |  |  |

Notes: Educational dummy variables indicate the highest level of attainment. These educational dummy variables are: university or college degree - either at undergraduate or postgraduate level; other higher degree - which includes work-related, or vocational, higher education qualifications such as Higher National Diplomas (HND) and Higher National Certificates (HNC); A-levels – which are post-compulsory examinations taken at 18 to qualify for college or university entrance; O-levels/GCSE’s – which are schooling attainment qualifications taken at 16; other qualifications; and lastly, no formal qualifications. The variable ‘number of sources for news’ counts the number of media sources the respondent uses to get their information about news and current affairs. Information on newspaper usage indicates the newspaper the respondent reads most frequently. These newspaper usage dummy variables are: doesn't read a newspaper; broadsheets – regarded as a more serious and less sensationalist outlet; redtop tabloids – regarded as sensationalist outlets which all have red mastheads; compact tabloids – regarded as sensationalist outlets; compact newspapers - a broadsheet-quality newspaper printed in a tabloid format; and lastly, regional newspapers. General health dummy variables indicate self-reported general health, where respondents are asked “In general, would you say your health is…”. Personality traits—Openness, Neuroticism, Extraversion, Conscientiousness and Agreeableness—are measured using the short 15-item Big-Five inventory (BFI-15). Each trait is based on a level of agreement with three statements, assessed on a seven-point scale. Responses are added across each set of the three statements and then divided by the number of items over which the sum is calculated.
