# Supplementary material for: Cognitive ability and voting behaviour in the 2016 UK referendum on European Union membership
Source: PLoS One. 2023 Nov 22;18(11):e0289312. doi: 10.1371/journal.pone.0289312 (PMC10664886; doi:10.1371/journal.pone.0289312)
Supplement: S2 Table — (DOCX) [file pone.0289312.s002.docx]

**Table S2.** **Pooled, multilevel and conditional (fixed-effect) logistic regressions measuring the relationship between cognitive ability and voting behaviour in the referendum.**

|  | (1) | (2) | (3) | (4) |
| --- | --- | --- | --- | --- |
| Dependent variable: | Voted Remain | Voted Remain | Voted Remain | Voted Remain |
| Regression: | Pooled  logistic | Multilevel logistic | Multilevel logistic | Conditional logistic |
|  |  |  |  |  |
| Cognitive Ability | 0.055*** | 0.037*** | 0.054*** | 0.109*** |
|  | [6.376] | [5.567] | [7.495] | [2.600] |
| Spouse Cognitive Ability |  |  | 0.043*** |  |
|  |  |  | [6.003] |  |
| Age (years) | -0.024 | -0.006 | -0.007 | 0.082 |
|  | [-0.888] | [-0.280] | [-0.316] | [0.449] |
| Age squared (years) | 0.000 | 0.000 | 0.000 | -0.002 |
|  | [0.825] | [0.196] | [0.223] | [-0.570] |
| Age cubed (years) | -0.000 | -0.000 | -0.000 | 0.000 |
|  | [-0.928] | [-0.332] | [-0.350] | [0.577] |
| Male | -0.022* | -0.032*** | -0.026** | -0.210*** |
|  | [-1.846] | [-2.896] | [-2.330] | [-3.483] |
| White | -0.127*** | -0.093*** | -0.108*** | -0.207 |
|  | [-3.913] | [-3.304] | [-3.854] | [-1.295] |
| University or college degree | 0.170*** | 0.135*** | 0.122*** | 0.174 |
|  | [4.608] | [4.409] | [4.012] | [0.871] |
| Other higher degree | 0.063 | 0.035 | 0.026 | -0.140 |
|  | [1.634] | [1.130] | [0.852] | [-0.694] |
| A-Level | 0.017 | 0.017 | 0.012 | -0.044 |
|  | [0.441] | [0.577] | [0.413] | [-0.241] |
| GCSE's | -0.029 | -0.024 | -0.030 | -0.195 |
|  | [-0.743] | [-0.823] | [-1.045] | [-1.092] |
| Other qualification | -0.007 | -0.017 | -0.022 | -0.218 |
|  | [-0.162] | [-0.550] | [-0.714] | [-1.086] |
| Self-employed | -0.010 | -0.003 | -0.008 | -0.102 |
|  | [-0.389] | [-0.136] | [-0.373] | [-0.851] |
| Unemployed | 0.106* | 0.071 | 0.065 | 0.042 |
|  | [1.823] | [1.337] | [1.246] | [0.107] |
| Full-time education | 0.216* | 0.148* | 0.138 |  |
|  | [1.658] | [1.653] | [1.529] |  |
| Retired | 0.015 | 0.034 | 0.027 | 0.320* |
|  | [0.501] | [1.435] | [1.121] | [1.906] |
| Economically inactive | 0.015 | -0.020 | -0.021 | -0.201 |
|  | [0.439] | [-0.737] | [-0.772] | [-1.296] |
| Interview mode: Telephone | -0.008 | -0.009 | -0.006 | 0.047 |
|  | [-0.170] | [-0.242] | [-0.153] | [0.178] |
| Interview mode: Web | 0.012 | 0.009 | 0.007 | 0.001 |
|  | [0.529] | [0.470] | [0.345] | [0.005] |
| Number of sources for news | 0.014*** | 0.006* | 0.005 | -0.026 |
|  | [3.048] | [1.685] | [1.515] | [-1.161] |
| Broadsheets | 0.077*** | 0.088*** | 0.080*** | 0.272** |
|  | [3.229] | [4.277] | [3.947] | [2.120] |
| Redtop tabloids | -0.141*** | -0.107*** | -0.097*** | -0.154 |
|  | [-4.675] | [-4.476] | [-4.095] | [-1.049] |
| Compact tabloids | -0.209*** | -0.134*** | -0.132*** | 0.004 |
|  | [-7.721] | [-6.163] | [-6.091] | [0.030] |
| Compact newspapers | 0.043 | 0.050 | 0.045 | 0.214 |
|  | [0.578] | [0.817] | [0.759] | [0.454] |
| Regional newspapers | 0.003 | 0.023 | 0.025 | 0.208 |
|  | [0.109] | [0.962] | [1.050] | [1.480] |
| Conservative | -0.005 | -0.004 | -0.005 | 0.141 |
|  | [-0.207] | [-0.218] | [-0.269] | [1.220] |
| Labour | 0.211*** | 0.172*** | 0.169*** | 0.385*** |
|  | [9.914] | [8.824] | [8.673] | [2.664] |
| Liberal | 0.252*** | 0.206*** | 0.201*** | 0.435** |
|  | [10.372] | [8.393] | [8.087] | [2.464] |
| Green | 0.193*** | 0.157*** | 0.151*** | 0.129 |
|  | [5.936] | [4.654] | [4.393] | [0.375] |
| Nationalist | -0.240*** | -0.171*** | -0.165*** | -0.252* |
|  | [-8.264] | [-6.419] | [-6.286] | [-1.708] |
| Other political party | 0.039 | 0.093 | 0.090 |  |
|  | [0.272] | [1.043] | [1.012] |  |
| General health: Excellent | 0.066 | -0.000 | -0.001 | -0.365 |
|  | [1.250] | [-0.012] | [-0.028] | [-1.418] |
| General health: Very good | 0.049 | -0.007 | -0.005 | -0.319 |
|  | [0.948] | [-0.183] | [-0.149] | [-1.256] |
| General health: Good | 0.017 | -0.023 | -0.020 | -0.327 |
|  | [0.327] | [-0.658] | [-0.561] | [-1.318] |
| General health: Fair | -0.014 | -0.050 | -0.047 | -0.455* |
|  | [-0.260] | [-1.376] | [-1.330] | [-1.716] |
| Long-term health condition | -0.012 | -0.022 | -0.021 | -0.168** |
|  | [-0.653] | [-1.563] | [-1.546] | [-2.065] |
| Openness | 0.003 | 0.004 | 0.003 | -0.012 |
|  | [0.385] | [0.671] | [0.496] | [-0.333] |
| Neuroticism | 0.022*** | 0.014** | 0.013** | 0.026 |
|  | [2.752] | [2.291] | [2.114] | [0.769] |
| Extraversion | 0.005 | -0.002 | -0.002 | -0.036 |
|  | [0.618] | [-0.370] | [-0.306] | [-1.038] |
| Conscientious | -0.010 | -0.007 | -0.007 | 0.018 |
|  | [-1.189] | [-1.110] | [-1.113] | [0.499] |
| Agreeableness | 0.021*** | 0.012* | 0.012* | 0.032 |
|  | [2.624] | [1.843] | [1.862] | [0.887] |
| Log of OECD household income (deflated) | 0.116*** | 0.128*** | 0.108*** |  |
|  | [5.150] | [6.150] | [5.234] |  |
| Married | 0.040 | 0.037 | 0.033 |  |
|  | [1.292] | [1.440] | [1.279] |  |
| Number of children in household | -0.004 | 0.002 | -0.004 |  |
|  | [-0.261] | [0.101] | [-0.287] |  |
| Square root of household size | 0.071 | 0.065 | 0.074 |  |
|  | [1.407] | [1.401] | [1.595] |  |
| Own house outright | 0.167*** | 0.173*** | 0.142*** |  |
|  | [3.386] | [3.830] | [3.131] |  |
| Own house with mortgage | 0.098** | 0.109** | 0.084* |  |
|  | [1.993] | [2.405] | [1.869] |  |
| Private sector renter | 0.080 | 0.100* | 0.072 |  |
|  | [1.375] | [1.754] | [1.235] |  |
| Financial decision maker: Spouse | -0.020 | -0.003 | -0.015 |  |
|  | [-0.778] | [-0.140] | [-0.739] |  |
| Financial decision maker: Equal | 0.031 | 0.031* | 0.024 |  |
|  | [1.337] | [1.672] | [1.291] |  |
| Financial decision maker: Other | -0.212 | -0.167 | -0.155 |  |
|  | [-1.163] | [-1.019] | [-0.985] |  |
| Lives in urban area | -0.007 | -0.001 | -0.000 |  |
|  | [-0.319] | [-0.060] | [-0.013] |  |
| North West | -0.029 | -0.027 | -0.025 |  |
|  | [-0.573] | [-0.590] | [-0.550] |  |
| Yorkshire and Humber | 0.033 | 0.020 | 0.026 |  |
|  | [0.631] | [0.399] | [0.527] |  |
| East Midlands | -0.023 | -0.030 | -0.031 |  |
|  | [-0.438] | [-0.628] | [-0.650] |  |
| West Midlands | -0.008 | -0.020 | -0.020 |  |
|  | [-0.161] | [-0.411] | [-0.422] |  |
| East of England | -0.009 | -0.014 | -0.015 |  |
|  | [-0.175] | [-0.310] | [-0.330] |  |
| London | 0.005 | 0.018 | 0.029 |  |
|  | [0.096] | [0.348] | [0.546] |  |
| South East | 0.010 | 0.001 | -0.001 |  |
|  | [0.218] | [0.025] | [-0.020] |  |
| South West | -0.029 | -0.025 | -0.028 |  |
|  | [-0.592] | [-0.557] | [-0.633] |  |
| Wales | 0.085* | 0.065 | 0.071 |  |
|  | [1.673] | [1.226] | [1.342] |  |
| Scotland | 0.256*** | 0.241*** | 0.236*** |  |
|  | [7.077] | [6.796] | [6.613] |  |
| Northern Ireland | 0.089 | 0.058 | 0.041 |  |
|  | [1.511] | [0.951] | [0.673] |  |
|  |  |  |  |  |
|  |  |  |  |  |
| Number of individuals | 6,366 | 6,366 | 6,366 | 926 |
| Number of households | 3,183 | 3,183 | 3,183 | 463 |
| Additional controls | Yes | Yes | Yes | Yes |
| Household random effects | No | Yes | Yes | No |
| Household fixed effects | No | No | No | Yes |
| Mean dependent variable | 0.566 | 0.566 | 0.566 | 0.500 |

Notes: Main entries are marginal effects in Column's 1, 2 and 3, and average semi-elasticates in Column 4, t-statistics in square brackets. Standard errors are clustered at the household to control for intra household correlations. Cognitive ability and personality traits—Openness, Neuroticism, Extraversion, Conscientiousness and Agreeableness—are all standardized for ease of interpretation. Significance levels *** 1%,** 5%, * 10%.
