# Supplementary material for: Cognitive ability and voting behaviour in the 2016 UK referendum on European Union membership
Source: PLoS One. 2023 Nov 22;18(11):e0289312. doi: 10.1371/journal.pone.0289312 (PMC10664886; doi:10.1371/journal.pone.0289312)
Supplement: S3 Table — (DOCX) [file pone.0289312.s003.docx]

**Table S3.** **Pooled, multilevel and fixed-effect linear regressions measuring the relationship between cognitive ability and voting behaviour in the referendum.**

|  | (1) | (2) | (3) | (4) |
| --- | --- | --- | --- | --- |
| Dependent variable: | Voted Remain | Voted Remain | Voted Remain | Voted Remain |
| Regression: | Pooled linear | Multilevel linear | Multilevel linear | Fixed-effect linear |
| *Actor:* |  |  |  |  |
| Cognitive Ability | 0.042*** | 0.029*** | 0.046*** | 0.014** |
|  | [6.413] | [5.559] | [7.798] | [2.362] |
| Word Recall | 0.025*** | 0.018*** | 0.031*** | 0.007 |
|  | [4.405] | [3.900] | [5.742] | [1.446] |
| Verbal Fluency | 0.016*** | 0.009* | 0.017*** | -0.000 |
|  | [2.647] | [1.773] | [3.191] | [-0.038] |
| Subtraction Test | 0.015** | 0.011** | 0.018*** | 0.008 |
|  | [2.485] | [2.519] | [3.114] | [1.604] |
| Fluid Reasoning | 0.027*** | 0.020*** | 0.034*** | 0.012** |
|  | [4.557] | [4.220] | [5.962] | [2.423] |
| Numerical Reasoning | 0.034*** | 0.020*** | 0.039*** | 0.008 |
|  | [5.304] | [3.790] | [6.474] | [1.359] |
| *Partner:* |  |  |  |  |
| Cognitive Ability |  |  | 0.036*** |  |
|  |  |  | [6.174] |  |
| Word Recall |  |  | 0.025*** |  |
|  |  |  | [4.643] |  |
| Verbal Fluency |  |  | 0.019*** |  |
|  |  |  | [3.529] |  |
| Subtraction Test |  |  | 0.012** |  |
|  |  |  | [1.993] |  |
| Fluid Reasoning |  |  | 0.026*** |  |
|  |  |  | [4.513] |  |
| Numerical Reasoning |  |  | 0.036*** |  |
|  |  |  | [6.179] |  |
|  |  |  |  |  |
| Number of individuals | 6,366 | 6,366 | 6,366 | 926 |
| Number of households | 3,183 | 3,183 | 3,183 | 463 |
| Additional controls | Yes | Yes | Yes | Yes |
| Household random effects | No | Yes | Yes | No |
| Household fixed effects | No | No | No | Yes |
| Mean dependent variable | 0.566 | 0.566 | 0.566 | 0.500 |

Notes: In all columns the main entries are marginal effects with t-statistics in square brackets. Standard errors are clustered at the household to control for intra household correlations. All columns include additional controls for age (cubic); gender; ethnicity, education; labour force status; interview mode; the number of sources used for information about news and current affairs; type of newspaper used for information about news and current affairs; political party supports/most aligned to; self-assessed general health; whether respondent suffers from long term health problem; and personality traits—Openness, Neuroticism, Extraversion, Conscientiousness, Agreeableness—which are measured using the short 15-item Big-Five inventory (BFI-15). Columns 1, 2 and 3 also includes further controls for household specific factors including the logarithm of monthly household income (adjusted by the OECD-modified equivalence scale and deflated by the Consumer Price Index); marital status; number of dependent children in the household; the square root of household size; housing tenure; household financial decision maker; whether lives in urban location; and region of residence dummy variables. Significance levels *** 1%,** 5%, * 10%.
