# Supplementary material for: Cognitive ability and voting behaviour in the 2016 UK referendum on European Union membership
Source: PLoS One. 2023 Nov 22;18(11):e0289312. doi: 10.1371/journal.pone.0289312 (PMC10664886; doi:10.1371/journal.pone.0289312)
Supplement: S4 Table — (DOCX) [file pone.0289312.s004.docx]

**Table S4.** **Multiverse estimation: Pooled, multilevel and fixed-effect linear regressions measuring the relationship between cognitive ability and voting behaviour in the referendum.**

|  | (1) | (2) | (3) | (4) |
| --- | --- | --- | --- | --- |
| Dependent variable: | Voted Remain | Voted Remain | Voted Remain | Voted Remain |
| Regression: | Pooled linear | Multilevel linear | Multilevel linear | Fixed-effect linear |
| *Actor:* |  |  |  |  |
| Cognitive Ability | 0.063 | 0.037 | 0.062 | 0.013 |
|  | [100, 100] | [100, 100] | [100, 100] | [100, 63] |
| Word Recall | 0.043 | 0.025 | 0.046 | 0.010 |
|  | [100, 100] | [100, 100] | [100, 100] | [100, 50] |
| Verbal Fluency | 0.026 | 0.012 | 0.026 | -0.0004 |
|  | [100, 97] | [100, 63] | [100, 100] | [63, 0] |
| Subtraction Test | 0.023 | 0.014 | 0.026 | 0.007 |
|  | [100, 95] | [100, 85] | [100, 100] | [100, 17] |
| Fluid Reasoning | 0.045 | 0.026 | 0.050 | 0.012 |
|  | [100, 100] | [100, 100] | [100, 100] | [100, 63] |
| Numerical Reasoning | 0.051 | 0.025 | 0.055 | 0.005 |
|  | [100, 100] | [100, 100] | [100, 100] | [83, 18] |
| *Partner:* |  |  |  |  |
| Cognitive Ability |  |  | 0.052 |  |
|  |  |  | [100, 100] |  |
| Word Recall |  |  | 0.038 |  |
|  |  |  | [100, 100] |  |
| Verbal Fluency |  |  | 0.028 |  |
|  |  |  | [100, 100] |  |
| Subtraction Test |  |  | 0.021 |  |
|  |  |  | [100, 100] |  |
| Fluid Reasoning |  |  | 0.042 |  |
|  |  |  | [100, 100] |  |
| Numerical Reasoning |  |  | 0.053 |  |
|  |  |  | [100, 100] |  |
|  |  |  |  |  |
| Number of individuals | 6,366 | 6,366 | 6,366 | 926 |
| Number of households | 3,183 | 3,183 | 3,183 | 463 |
| Additional controls | Yes | Yes | Yes | Yes |
| Household random effects | No | Yes | Yes | No |
| Household fixed effects | No | No | No | Yes |
| Mean dependent variable | 0.566 | 0.566 | 0.566 | 0.500 |

Notes: All columns include additional controls for age (cubic); gender; ethnicity, education; labour force status; interview mode; the number of sources used for information about news and current affairs; type of newspaper used for information about news and current affairs; political party supports/most aligned to; self-assessed general health; whether respondent suffers from long term health problem; and personality traits—Openness, Neuroticism, Extraversion, Conscientiousness, Agreeableness—which are measured using the short 15-item Big-Five inventory (BFI-15). Columns 1, 2 and 3 also includes further controls for household specific factors including the logarithm of monthly household income (adjusted by the OECD-modified equivalence scale and deflated by the Consumer Price Index); marital status; number of dependent children in the household; the square root of household size; housing tenure; household financial decision maker; whether lives in urban location; and region of residence dummy variables. Following Young and Holsteen [58], the main entries are marginal effects representing the mean estimate across 128 unique combinations of control variables. In square brackets we report sign stability (%) and statistical significance rate (%) for our estimates across the 128 unique combinations of control variables, with $p< .05$ being the threshold for statistical significance. For example, [100, 100] would indicate that with these 128 unique combinations of control variables, it is not possible to find an opposite signed or even nonsignificant estimate.
