# Supplementary material for: Cognitive ability and voting behaviour in the 2016 UK referendum on European Union membership
Source: PLoS One. 2023 Nov 22;18(11):e0289312. doi: 10.1371/journal.pone.0289312 (PMC10664886; doi:10.1371/journal.pone.0289312)
Supplement: Attachment — Submitted filename: Observations and comments.docx [file pone.0289312.s005.docx]

Observations and comments

The paper titled; “Cognitive Ability and Voting Behaviour in the 2016 UK Referendum on European Union Membership” uses nationally representative data and a barrage of control variables to assess the link between cognitive function and voting behavior in the UK’s 2016 referendum on its membership in the EU. The findings are plausible and challenge the ideas that political behaviors of voters are solely a function of socioeconomic issues. The analysis is based on the premise that the period leading up to the referendum was characterized by a significant volume of misinformation and disinformation and it may affect the voter’s behavior. Moreover, it is obvious that higher cognitive ability and analytical thinking increase the propensity to detect and resist misinformation. Below are some of my observations. I could not understand why the author used cognitive ability and its constructs in the regression equations simultaneously. Would it not be causing multicollinearity problem? Moreover, I think cognitive ability measures may be highly subject to biasedness due to education level and discipline of the respondent. The results discussion is too brief and can be elaborated to make it clearer for the reader. It may improve the usefulness of the findings considerably.
