# Supplementary material for: Cognitive ability and voting behaviour in the 2016 UK referendum on European Union membership
Source: PLoS One. 2023 Nov 22;18(11):e0289312. doi: 10.1371/journal.pone.0289312 (PMC10664886; doi:10.1371/journal.pone.0289312)
Supplement: Attachment — Submitted filename: PONE_D_23_06300_RESPONSE to REVIEWERS.docx [file pone.0289312.s006.docx]

**ACADEMIC EDITOR____________________________________________________________________**

*Thank you for the opportunity to revise and resubmit our paper toward potential publication in PLOS ONE. We have carefully read and responded to all of the comments as discussed below. We believe that the paper has substantially improved as a result of this process and thank yourself and the two reviewers for your time in this.*

1. Please ensure that your manuscript meets PLOS ONE's style requirements, including those for file naming.

*Author’s Response: Thank you for the opportunity to revise and resubmit our paper to PLOS ONE. As part of the revision process, we have carefully revised the paper to be in accordance with PLOS ONE’s style requirements.*

2. In your Data Availability statement, you have not specified where the minimal data set underlying the results described in your manuscript can be found. PLOS defines a study's minimal data set as the underlying data used to reach the conclusions drawn in the manuscript and any additional data required to replicate the reported study findings in their entirety. All PLOS journals require that the minimal data set be made fully available. For more information about our data policy, please see http://journals.plos.org/plosone/s/data-availability.

"Upon re-submitting your revised manuscript, please upload your study’s minimal underlying data set as either Supporting Information files or to a stable, public repository and include the relevant URLs, DOIs, or accession numbers within your revised cover letter. For a list of acceptable repositories, please see http://journals.plos.org/plosone/s/data-availability#loc-recommended-repositories. Any potentially identifying patient information must be fully anonymized.

*Author’s Response: Thank you for bringing this requirement to our attention. We have now included the Data Availability details at the beginning of the paper.*

3. Please include captions for your Supporting Information files at the end of your manuscript, and update any in-text citations to match accordingly.

*Author’s Response: Thank you for bringing this requirement to our attention. We have now revised the paper accordingly.*

**REVIEWER 1**

*We very much appreciate your comments.*

*The paper has been considerably revised and we respond to your specific comments below.*

Reviewer #1: 1. It's 2023 now. What's the point of the author studying the UK's vote to leave the European Union in 2016? What are the effects of these conclusions and research findings on the present?

*Author response: This is a good point, and we agree that we need to make the importance of our research findings clearer. In this respect, it is worth noting that there have been 58 referendums on European Union matters alone, since 1972^1^. Switzerland – as a semi-direct democracy - has an average of four referendums per year^2^. Referendums have always been controversial as they are a trade-off between the direct engagement of citizens in deciding a political outcome and a lack of refinement and deliberation of the issue through elected representatives within the checks and balances of established political institutions. Indeed, there is an academic literature in its own right which debates the merits (directly acting on the will of the people) and potential concerns (the ability of the electorate to decide a technically complex issue in isolation) of referendums. See Clark (1998) and Frey (1994) for contrasting examples of this debate. We add to this debate by showing that the outcome of referendums can be influenced by the inability of the less cognitively able to identify mis/disinformation. Going forward, for future referendums, such as a potential future Scottish Independence referendum, governments and policy makers should legislate accordingly.*

*To make this point, we have now added a paragraph to the Introduction which speaks to all of the above.*

*^1^	Del Monet, M. (2022). Referendums on EU issues. Fostering civic engagement. European Parliamentary Research Services. https://www.europarl.europa.eu/RegData/etudes/IDAN/2022/729358/EPRS_IDA(2022)729358_EN.pdf*

*^2^ Federal Chancellory BK. Referendums. Schweizerische Eidgenossenschaft. https://www.bk.admin.ch/bk/de/home/politische-rechte/volksabstimmungen.html*

*^3^* *Clark, S. J. (1998). A Populist Critique of Direct Democracy. Harvard Law Review, 112(2), 434–482.*

*Frey, B. S. (1994). Direct Democracy: Politico-Economic Lessons from Swiss Experience. The American Economic Review, 84(2), 338–342.*

2. In the fifth line of the second paragraph of the introduction, more than three authors, just list the first author and add et al. There are similar problems in the content behind the manuscript, the author himself checked it.

*Author response: Thank you for reminding us of this convention. It is also the one that we ordinarily would use (and should have done for our initial submission). However, we have received the PLOS ONE Publishing Style requirements from the Associate Editor which includes the use the Numbered Citation method, which we have now converted the manuscript to adhere to.*

3. At the end of the introduction, the author puts forward the innovation of the manuscript, but the innovation is too little, so it is suggested to supplement it.

*Author response: Thank you for another helpful comment. We have now revisited this paragraph and succinctly summarised the key innovations of our work as compared to the previous literature. We believe that the Reader should now be able to better understand the contribution of our work.*

4. The footnote reference format of 4.2.1 is not correct.

*Author response: Thank you for spotting this. We have now revised the paper to be in accordance with all of the Style Requirements of PLOS ONE (including References) and proofread the paper multiple times to correct for any other typos.*

5. 5.2.2 Whether the title can be considered as the independent variable.

*Author response: Thank you for pointing out the need for consistency in the subsection titles. We have now changed this to ‘Independent Variable’ as you suggested.*

6. The author's empirical results have achieved relatively good results, but there is no robustness test, and a robustness test needs to be added to prove that the results obtained by the author are robust.

*Author response: Thank you for this comment. We agree that we need some further robustness checks. Section 3.2 in the manuscript is now dedicated to robustness checks. Firstly, we check for non-linearities in the relationship between cognitive ability and voting Remain (Figure 3). Secondly, we test whether our results derived from binary choice models are robust to the use of linear probabilities models (Table S3 in the Supporting Information), which then allows us to implement our final strategy, which is to examine how our cognitive ability coefficients behave when the regression specification is modified by adding or removing regressors (Table S4 in the Supporting Information). In the final robustness check mentioned above, we adopt the approach proposed by Young and Holsteen (2017), where our analysis is conducted across 128 unique combinations of control variables. Here, the reporting of coefficients reflects the mean estimate from the 128 unique combinations. The results of this procedure suggest our main findings are robust to combinations of control variables, and therefore do not depend on knife-edge specifications (i.e., a specific combination of regressors).*

*Young, C., & Holsteen, K. (2017). Model uncertainty and robustness: A computational framework for multimodel analysis. Sociological Methods & Research, 46(1), 3-40.*

7. The representation of the percentage in the manuscript is recommended to be unified. In some places, the symbol “%” is used, and in some places, the “percentage points” is used.

*Author response: Thank you for this comment. To clarify, the difference in presentation is intentional when describing a unit increase change (e.g., 10% to 15% would be described as a 5-percentage point increase) versus a relative increase (to continue the example, a 50% increase). This depends on what it is we are trying to emphasise in the discussion.*

**REVIEWER 2**

*We very much appreciate your comments.*

*The paper has been considerably revised and we respond to your specific comments below.*

Reviewer #2: The paper titled; “Cognitive Ability and Voting Behaviour in the 2016 UK Referendum on European Union Membership” uses nationally representative data and a barrage of control variables to assess the link between cognitive function and voting behavior in the UK’s 2016 referendum on its membership in the EU. The findings are plausible and challenge the ideas that political behaviors of voters are solely a function of socioeconomic issues. The analysis is based on the premise that the period leading up to the referendum was characterized by a significant volume of misinformation and disinformation and it may affect the voter’s behavior. Moreover, it is obvious that higher cognitive ability and analytical thinking increase the propensity to detect and resist misinformation. Below are some of my observations.

1. I could not understand why the author used cognitive ability and its constructs in the regression equations simultaneously. Would it not be causing multicollinearity problem?

*Author response: To clarify, we do not use cognitive ability and its constructs in the regression equations simultaneously, as you correctly note this would cause multicollinearity problems. We have now clarified this in our description of the results to make this clearer to the Reader.*

2. Moreover, I think cognitive ability measures may be highly subject to biasedness due to education level and discipline of the respondent.

*Author response: Thank you for this comment. On the one hand, we do control for the individual’s level of education as previously shown to be an important predictor of a vote to Remain versus Leave.^1^ On the other hand, educational attainment and cognitive ability are fundamentally interconnected.^2^ To address this point, we have now added the following discussion within the last paragraph of Section 3.1 Main Results:*

*“This is additionally interesting as educational attainment and cognitive ability are fundamentally interconnected (Malanchini et al. 2020). Education is an important control owing to the tendency for lower cognitive ability to be associated with lower levels of education, which is associated—perhaps because less educated voters are those who might find it harder to understand the opportunities from globalization that accompanies EU membership—with the tendency to vote Leave (Becker et al., 2017). Whilst we control for educational attainment, it is possible that the type of university attended or the subject of study may be correlated with both cognitive ability and a tendency to vote Remain, leading to a bias in our parameter estimates. Nevertheless, even after controlling for educational attainment and a host of other covariates, the observed positive relationship between cognitive ability and a Remain vote is extremely strong. This suggests that the likelihood of an unobservable confounder, such as the subject of study, accounting for the entirety of the estimated relationship between cognitive ability and a vote to Remain in the EU is rather low.*

*^1^	Becker, S.O., Fetzer, T, & Novy D. (2017). Who voted for Brexit? A comprehensive district level analysis. Economic Policy, 32 (92), 601-650.*

*^2^	Malanchini, M., Rimfeld, K., Allegrini, A. G., Ritchie, S. J., & Plomin, R. (2020). Cognitive ability and education: How behavioural genetic research has advanced our knowledge and understanding of their association. Neuroscience and biobehavioral reviews, 111, 229–245.*

3. The results discussion is too brief and can be elaborated to make it clearer for the reader. It may improve the usefulness of the findings considerably.

*Author response: We have now expanded the results section in three distinct ways. Firstly, we make it clear throughout the results section that we do not use cognitive ability and its constructs in the regression equations simultaneously. Secondly, we extend the explanation of the important link between education and cognitive ability in explaining voting behaviour. Thirdly, we include a series of robustness checks, as suggested by another Reviewer. Specifically, we check for non-linearities in the relationship between cognitive ability and voting Remain (Figure 3). We then test whether our results derived from binary choice models are robust to the use of linear probabilities models (Table S3 in the Supporting Information), which then allows us to implement our final strategy, which is to examine how our cognitive ability coefficients behave when the regression specification is modified by adding or removing regressors (Table S4 in the Supporting Information). In the final robustness check mentioned above, we adopt the approach proposed by Young and Holsteen (2017), where our analysis is conducted across 128 unique combinations of control variables. Here, the reporting of coefficients reflects the mean estimate from the 128 unique combinations. The results of this procedure suggest our main findings are robust to combinations of control variables, and therefore do not depend on knife-edge specifications (i.e., a specific combination of regressors).*

*Young, C., & Holsteen, K. (2017). Model uncertainty and robustness: A computational framework for multimodel analysis. Sociological Methods & Research, 46(1), 3-40.*
