# Supplementary material for: Cognitive ability and voting behaviour in the 2016 UK referendum on European Union membership
Source: PLoS One. 2023 Nov 22;18(11):e0289312. doi: 10.1371/journal.pone.0289312 (PMC10664886; doi:10.1371/journal.pone.0289312)
Supplement: Attachment — Submitted filename: PONE_D_23_06300_RESPONSE to REVIEWERS.docx [file pone.0289312.s007.docx]

**ACADEMIC EDITOR____________________________________________________________________**

*Thank you for the opportunity to revise and resubmit our paper toward potential publication in PLOS ONE. We have carefully read and responded to all of the comments as discussed below.*

**REVIEWER 1**

*We very much appreciate your comments.*

*We respond to your specific comment below.*

Reviewer #1: The author has been modified according to the opinions put forward, and has answered it very carefully. Finally, it is suggested that the limitations and future prospects of the study should be added at the end of the article.

We have a paragraph on limitations at the end of the article, although we do now include a suggestion for a future area of research.

**REVIEWER 2**

Reviewer #2: The manuscript has been improved significantly and all the comments are incorporated to my satisfaction.

*Thank you.*

**REVIEWER 3**

*We very much appreciate your comments.*

*We respond to your specific comments below.*

Reviewer #3: Thanks, interesting manuscript. I was invited to the second round of reviews.
This is a professionally prepared manuscript, the methods are good and the topic is relevant.
I only have three smaller but important suggestions, all of them should be implemented. Plus some_minor.

(1) If I put it polemically: The message of the study is that a) being for Brexit is stupid and that b) the people who voted for Brexit are stupid. Stupid + stupid = super stupid. It also becomes clear what opinion the authors themselves represent. To moderate the whole thing rhetorically, I would mention a few names of prominent people who were in favor of Brexit, right at the beginning. For example: “The issue of Brexit was very controversial among the British public. Well-known celebrities like Mick Jagger (+ more names) were for it, others (a few names) were against it.”

*Thank you for this suggestion, we do want to give a balanced view despite the clear empirical evidence. We follow your suggestion and mention some high-profile celebrities and politicians who backed Brexit very early on in the Introduction section.*

(2) Absolutely: Present a correlation table with correlations (r between –1, 0 and +1) between all important variables, most importantly: between intelligence and Brexit voting behavior.

*Thank you for this suggestion, we now include a correlation matrix which shows the correlations between the dependent variable, independent variable and a selection of the key control variables used in the study.*

(3) Add the IQ scores of voters for and against Brexit and the IQ difference. If you don't have these numbers, then on a small z scale. What is the difference between the two in SD units?

*Thank you for this suggestion, we now include Figure 2 which reports the distributions of our composite measure of cognitive ability, which is standardized for ease of interpretation, for those who voted Remain or Leave in the referendum. We also, as you suggest, report the mean scores of the voters and difference in SD units. Specifically, we include:*

*“The mean of the Leave (Remain) voters cognitive ability distribution is -0.245 (0.188) which suggests that the average Leave (Remain) voter falls below (above) the population average. The difference in means between voter types is 0.433 of a standard deviation, with a t-test confirming the difference is statistically significant,* $t=17.5, p<0.001$*.”*

Abstract:
Add for this sentence ”We find that higher cognitive ability is strongly linked to a preference for Remain at the individual level ([Pearson/Spearman] r=.??), that partner’s cognitive ability influences the respondent’s preferences ([standardized] beta=.??) and lastly, that having a cognitive ability advantage over one’s partner increases the likelihood of voting Remain ([standardized] beta=.??).”

*Thank you for this suggestion, we agree it is useful to have effect sizes in abstracts. We decided to include %’s so all effects can be included in the same denomination.*

Introduction:
Intelligence also has a positive effect (at the country level) on considering the interests of others:
Rindermann, H. & Carl, N. (2020). The good country index, cognitive ability and culture. Comparative Sociology, 19, 39–68.

*Thank you for bringing this reference to our attention. As suggested, we have now added this as additional evidence, in the Introduction, on the relationship between cognitive ability and personality traits.*

Results:
Figure 1 would be also possible as a scatterplot? (Not only bar chart) Add the Pearson/Spearman correlation in the notes of Figure 1.

*We think it would be unusual to have a scatterplot for a binary and continuous variable. What would be more common would be to have a binned scatter plot, which groups the x-axis variable (cognitive ability) into equal-sized bins, then compute the mean of the y-axis variable (Remain/Leave Vote) within each bin, and then create a scatterplot of these data points. This is analogous to what Figure 1 does, specifically it splits cognitive ability into 10 equal-sized bins and plots the fraction of those who voted Remain within each bin. We do however now include Figure 2 which shows the distribution of cognitive ability by voter type. The correlation between cognitive ability and voter type is now presented in the correlation matrix (Table 1).*

Good luck, this study will be well received.

*Thank you.*

**REVIEWER 4**

Reviewer #4: The author has responded to all of the feedback provided by reviewers. This paper is now ready for publication.

*Thank you.*
